# A New Folding Kinetic Mechanism for Human Transthyretin and the Influence of the Amyloidogenic V30M Mutation

**DOI:** 10.3390/ijms17091428

**Published:** 2016-08-31

**Authors:** Catarina S. H. Jesus, Zaida L. Almeida, Daniela C. Vaz, Tiago Q. Faria, Rui M. M. Brito

**Affiliations:** 1 Chemistry Department and Coimbra Chemistry Centre, Faculty of Science and Technology, University of Coimbra, Coimbra 3004-535, Portugal; catarina.jesus@student.qui.uc.pt (C.S.H.J.); zalmeida@qui.uc.pt (Z.L.A.); daniela.vaz@ipleiria.pt (D.C.V.); 2 Center for Neuroscience and Cell Biology, University of Coimbra, Coimbra 3004-504, Portugal; tfaria@qui.uc.pt; 3 Health Research Unit, School of Health Sciences, Leiria 2411-901, Portugal

**Keywords:** transthyretin, WT-TTR, V30M-TTR, folding kinetics, amyloid, FAP, ATTR

## Abstract

Protein aggregation into insoluble amyloid fibrils is the hallmark of several neurodegenerative diseases, chief among them Alzheimer’s and Parkinson’s. Although caused by different proteins, these pathologies share some basic molecular mechanisms with familial amyloidotic polyneuropathy (FAP), a rare hereditary neuropathy caused by amyloid formation and deposition by transthyretin (TTR) in the peripheral and autonomic nervous systems. Among the amyloidogenic TTR mutations known, V30M-TTR is the most common in FAP. TTR amyloidogenesis (ATTR) is triggered by tetramer dissociation, followed by partial unfolding and aggregation of the low conformational stability monomers formed. Thus, tetramer dissociation kinetics, monomer conformational stability and competition between refolding and aggregation pathways do play a critical role in ATTR. Here, we propose a new model to analyze the refolding kinetics of WT-TTR and V30M-TTR, showing that at pH and protein concentrations close to physiological, a two-step mechanism with a unimolecular first step followed by a second-order second step adjusts well to the experimental data. Interestingly, although sharing the same kinetic mechanism, V30M-TTR refolds at a much slower rate than WT-TTR, a feature that may favor the formation of transient species leading to kinetic partition into amyloidogenic pathways and, thus, significantly increasing the probability of amyloid formation in vivo.

## 1. Introduction

Amyloid formation has been associated with a wide range of pathologies [1,2], such as familial amyloidotic polyneuropathy (FAP) [3,4], Alzheimer’s disease (AD) [5], Parkinson’s disease (PD) [6], spongiform encephalopathies (SE) [7] and light-chain amyloidosis (AL) [8], the latter being the most common type of systemic amyloidosis. In amyloidosis, soluble proteins, which are innocuous in their native state, sample partially unfolded conformations and form cytotoxic soluble aggregates and, eventually, insoluble fibrils [1,2,9,10]. Transthyretin (TTR) is one of the more than forty different human peptides and proteins that have been identified in disease-related amyloid deposits [4,9,11] targeting different tissues and organs. To date, more than one hundred amyloidogenic TTR mutations have been identified leading to a variety of clinical manifestations ranging from peripheral and autonomic neuropathy, to cardiomyopathy, ophthalmopathy and vitreous opacities and carpal tunnel syndrome, among others [12,13,14,15,16,17,18,19,20,21]. One of the most common TTR mutations, V30M-TTR, is associated with peripheral and autonomic neuropathy at the early stages of the disease (FAP), a hereditary autosomal dominant lethal disease that may affect individuals from their twenties [14,18]. Although with higher incidence in Portugal, this TTR mutation is also prevalent in Sweden, Japan, Brazil and Majorca [19,20,21] and also found across many other countries and among different families without apparent genetic relation.

Human TTR is a homotetrameric protein, with a total molar mass of 55 kDa, found in the plasma, cerebrospinal fluid and eye [11,12,22,23]. It is mainly synthesized by the liver, but also by the choroid plexus and retinal pigment epithelium [11,12,22,23], and its main known functions are the transport of thyroxine (T_4_) and retinol, the latter in association with the retinol-binding protein (RBP). The concentration of TTR in the serum ranges from 3.1 to 7.6 μM, whereas in the cerebrospinal fluid varies between 0.09 and 0.36 μM [24]. The main structural element of each TTR subunit is a β-sandwich consisting of two β-sheets with four β-strands each. The association of the four subunits in the native tetramer forms a central channel with two thyroxine-binding sites [25], as seen in the crystal structures of WT- and V30M-TTR [26,27].

We have previously shown that amyloidogenic and non-amyloidogenic TTR variants dissociate to non-native monomers, in conditions close to physiological, with the amyloidogenic variants producing larger amounts of partially unfolded monomeric species, as a consequence of the marginal conformational stability of the non-native monomeric state, and able to sample conformations compatible with aggregation into amyloid. Moreover, the native tetrameric form of WT-TTR and its natural amyloidogenic variants V30M- and L55P-TTR, along with the non-amyloidogenic T119M-TTR are highly stable to thermal unfolding, with the tetrameric forms of the amyloidogenic variants having only slightly lower thermodynamic stabilities than the non-amyloidogenic T119M-TTR [11,28]. Thus, TTR tetramer dissociation kinetics, monomer conformational stability and the partitioning between refolding/reassembly of the tetramer versus aggregation pathways may play a critical role in amyloid formation in vivo [11,28,29,30,31,32,33,34,35,36].

In order to further investigate the kinetic partitioning of the TTR monomeric species between aggregation pathways leading to amyloid fibril formation and functional tetramer reassembling pathways, here we explore the refolding kinetics of WT-TTR and the amyloidogenic variant V30M-TTR. Previous refolding studies on WT-TTR, performed by Kelly and collaborators [37], were conducted over a relatively wide range of protein concentrations, and an unusual three-step refolding mechanism for tetramer assembly was proposed, based on the fluorescence variation of extrinsic probes. In the present work, low protein concentrations, close to physiological-like conditions, and monitoring of the intrinsic protein fluorescence were used to compare the refolding behavior of WT- and V30M-TTR and to determine the refolding kinetic constants of the two proteins.

It is commonly accepted that amyloid formation may result from unfolding-misfolding events in the extracellular milieu [1,2,9,10,11]. Therefore, longer refolding times can in fact favor the formation of non-native monomeric species in vivo and increase the probability of aggregate formation in the extracellular environment [2,10,11].

## 2. Results

In this work, we study the refolding process of WT-TTR and its amyloidogenic variant V30M-TTR from chemically-denatured monomers to native tetramers.

Preliminary refolding experiments from 2.0 M guanidinium thiocyanide (GdmSCN) showed that complete unfolding and refolding of TTR occurs in a very narrow GdmSCN concentration range (data not shown). Therefore, in order to study the refolding kinetics in a wider range of denaturant concentrations and at a larger range of apparent rate constants, protein unfolding was performed in two steps, as described in the Materials and Methods section. TTR was first incubated in 2.0 M GdmSCN for 12 h, followed by a 10,000-fold dialysis against 6.0 M urea during 10 h. Then, protein refolding experiments were performed by sample dilution to the desired final urea and protein concentrations. The refolding process of TTR was monitored by two methods: denaturant gradient gel electrophoresis (DGGE) and intrinsic fluorescence spectroscopy. While urea gradient gel electrophoresis experiments allowed us to qualitatively compare the kinetic profiles of WT- and V30M-TTR, the refolding experiments, followed by tryptophan emission, allowed the determination of apparent kinetic constants for the refolding process of WT- and V30M-TTR, at different urea concentrations.

### 2.1. The Presence of the V30M Mutation Influences the Refolding Profile of Transthyretin

**Figure 1:**
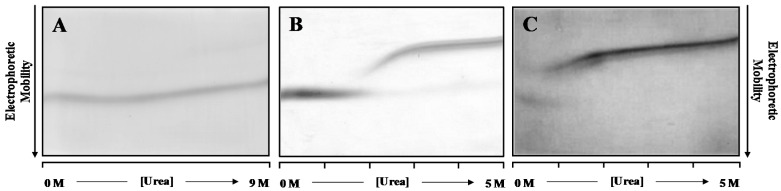
Denaturant gradient gel electrophoresis (DGGE) patterns of TTR (0.5 magnification factor). (**A**) Pattern for WT-TTR when a native sample is loaded onto the gel. Refolding patterns of WT-TTR (**B**) and V30M-TTR (**C**) when previously urea-unfolded samples are applied to the gel; TTR samples at approximately 1.0 µM were applied across the top of the gels containing a continuous urea gradient from left to right and submitted to electrophoresis at 20 mA during 4 h (**A**–**C**).

Patterns generated in urea gradient gel electrophoresis experiments depend on the thermodynamics and kinetics of urea-induced unfolding/refolding transitions [38,39,40,41]. Within the electrophoresis time scale, rapid transitions are described by continuous protein bands, with a single S-shaped curve and a single inflexion point in the transition zone; whereas, slow conformational interconversions are described by discontinuous bands [38,39,40,41]. Figure 1 shows the DGGE refolding patterns of WT- and V30M-TTR, when previously unfolded TTR is applied to a 0.0 to 5.0 M urea gradient gel. Analysis of Figure 1 allows the identification of two main protein bands in each gel. In an electrophoretic migration, the mobility of the sample is dictated by the charge, hydrodynamic volume, shape and the characteristics of the separation medium [38,42]. In the case of TTR and under the conditions tested, the electrophoretic mobility of the unfolded protein species is lower than the electrophoretic mobility of the folded globular protein. Hence, while upper bands correspond to unfolded TTR monomers, lower bands have been identified as belonging to refolded tetrameric TTR, because they exhibit similar electrophoretic mobility to the bands observed when native WT-TTR is applied onto the gel (Figure 1A). Moreover, in these conditions, TTR tetramers do not fully unfold even at high urea concentrations (9.0 M), on the time scale of the electrophoresis (4 h).

However, pre-incubation of WT- and V30M-TTR in 2.0 M GdmSCN followed by 6.0 M urea for a period of 22 h is enough to fully denature the TTR tetramers of WT- (Figure 1B) and V30M-TTR (Figure 1C), as judged by the single monomer band in the gels, at high molar urea concentrations. Furthermore, as shown in Figure 1B, refolding of WT-TTR occurs within the range of 1.5 to 3.0 M urea, with a transition with an apparent midpoint at approximately 2.2 M urea. The unfolded state predominates at high urea concentrations, and there is no indication of multiple unfolded states, as demonstrated by the constant mobility of the unique band of monomer across the gel. At urea concentrations around 3.0 M, the electrophoretic mobility of the WT-TTR band increases abruptly, forming a sigmoidal curve. This sharp transition indicates that the unfolded state is in rapid equilibrium with a more compact folded state. However, the electrophoretic mobility at the end of the S-shaped curve does not coincide with that of the refolded WT-TTR tetramer, observed at low urea concentrations (<2.0 M). At low urea concentrations, only a single band with a constant electrophoretic mobility is detected, which corresponds to the fully-refolded WT-TTR tetramer. These results show that the refolding pattern of WT-TTR is more complex than a simple two-state cooperative refolding transition and suggest the presence of an intermediate state. The refolding pattern of V30M-TTR (Figure 1C) is globally similar to that of WT-TTR. However, the apparent midpoint of the refolding transition occurs at lower urea concentrations than for WT-TTR. Likewise, the fully-refolded state of V30M-TTR presents similar electrophoretic mobility to that observed in the WT-TTR pattern, but it is only detected at urea concentrations lower than 1.2 M, reflecting the lower conformational stability of the amyloidogenic variant V30M-TTR [29].

DGGE experiments indicate that the refolding transitions occur within the range of 1.8 to 2.8 M urea for WT-TTR and of 0.4 to 1.2 M for V30M-TTR. Therefore, in order to perform a more detailed study of the refolding kinetics of TTR, we have studied the refolding behavior of the two proteins, WT- and V30M-TTR, in these ranges of urea concentrations, monitored by direct intrinsic protein fluorescence, at physiologically-relevant protein concentrations and experimental conditions.

### 2.2. Characterization of the Unfolded and Refolded Species

**Figure 2:**
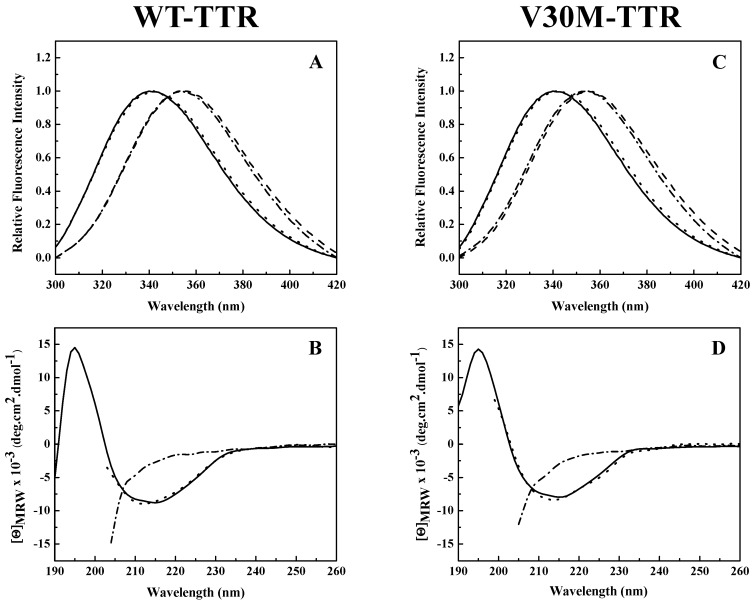
Conformational changes of WT-TTR and V30M-TTR upon unfolding and refolding. Fluorescence spectra (**A**,**C**) and CD spectra (**B**,**D**) of WT-TTR (**A**,**B**) and V30M-TTR (**C**,**D**) under native conditions (solid lines) (20 mM sodium phosphate buffer, 150 mM sodium chloride, pH 7.0), after incubation with 2.0 M GdmSCN for 12 h (dashed lines), followed by dialysis against 6.0 M urea for 10 h (dashed dotted lines) and after refolding upon extensive dialysis against sodium phosphate buffer (20 mM sodium phosphate buffer, 150 mM sodium chloride, pH 7.0) (dotted lines). Protein concentrations were 1.0 µM in the final refolding mixture. Fluorescence spectra were recorded with an excitation wavelength of 290 nm.

Analysis of the intrinsic tryptophan fluorescence spectra (Figure 2A,C) revealed that the native tetramers of WT- and V30M-TTR have very similar emission maxima, at approximately 340 nm, characteristic of partially-buried tryptophans. TTR has two tryptophan residues in each of its four identical subunits at positions 41 (Trp 41) and 79 (Trp 79). Trp 79 is located in the single α-helix of the protein positioned between β-strands E and F, while Trp 41 is located in the loop proximal to the beginning of β-strand C [25]. Previous studies showed that in the tetrameric form of the protein, while Trp 41 has a solvent exposure of 34.1%, Trp 79 is almost totally buried in the protein core and presents a solvent exposure of only 1.0% [34]. Moreover, it is known that the intrinsic fluorescence exhibited by TTR at pH 7.0 is mainly due to Trp 41 [33]. This agrees well with our results (Figure 2A,C).

A large red-shift in the emission maximum (>355 nm) occurs upon TTR unfolding in 2.0 M GdmSCN (Figure 2A,C), due to an increase in solvent exposure of the tryptophans and consequently a less hydrophobic environment around the tryptophan residues in the denatured state. Moreover, the fluorescence spectra of both TTR variants incubated in 2.0 M GdmSCN virtually overlap the spectra taken after dialysis against 6.0 M urea, indicating that urea is able to maintain the GdmSCN-induced denatured state (Figure 2A,C). The fluorescence spectra also show that the refolded species of WT- and V30M-TTR have emission spectra very similar to those of the native tetramers, with emission maxima of approximately 340 nm, indicating that protein refolding was achieved upon urea dilution. These results suggest that, upon refolding, tryptophan residues completely recover the solvent exposure characteristic of the native state.

The changes in secondary structure exhibited by WT- and V30M-TTR upon unfolding and refolding from urea were monitored by far-UV circular dichroism (CD). As shown in Figure 2B,D, both native TTR variants present a CD profile typical of mainly β-sheet proteins, with a single negative band, at approximately 213 nm, and a positive band around 195 nm. On the other hand, under urea denaturing conditions, the CD spectra of WT- and V30M-TTR show a negative band of large magnitude around 200 nm accompanied by a significant decrease in intensity at 213 nm, due to the loss of β-sheet structure (Figure 2B,D). Conversely, upon dilution of the samples from 6.0 M urea, the far-UV CD spectra of the refolded WT- and V30M-TTR virtually overlap with the spectra of the native proteins, with a single negative band at 213 nm, indicating that after urea dilution, the residual concentration of urea has a negligible effect on the secondary structure of the proteins (Figure 2B,D). These results show that both WT- and V30M-TTR remain largely unfolded in 6.0 M urea, following GdmSCN-induced unfolding, but the native-like secondary structure of WT- and V30M-TTR is completely recovered upon refolding from urea, demonstrating the reversibility of the process.

### 2.3. Transthyretin Reassembled Tetramers Show Native-Like Properties

**Figure 3:**
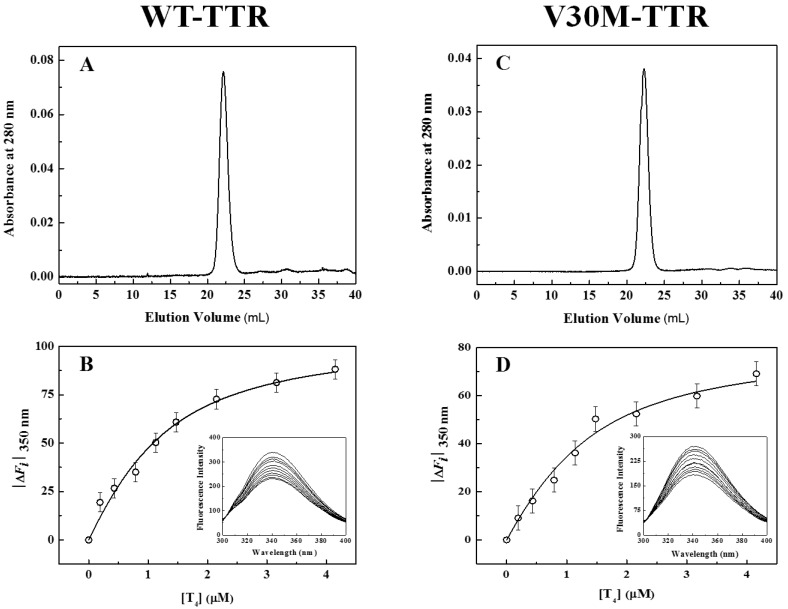
Characterization of the TTR refolded species. (**A**,**C**) Size-exclusion chromatograms of WT-TTR (**A**) and V30M-TTR (**C**) after complete protein refolding. The size-exclusion chromatography (SEC) experiments were run at a flow rate of 0.4 mL/min, in 20 mM sodium phosphate buffer, 150 mM sodium chloride, pH 7.0, at 25 °C; (**B**,**D**) Binding isotherm curves of thyroxine to refolded WT-TTR (**B**) and to refolded V30M-TTR (**D**) monitored by the variation of the intrinsic protein fluorescence emission at 350 nm. Protein concentrations were 1.0 μM in 20 mM sodium phosphate buffer, 150 mM sodium chloride, pH 7.0. Graph insets show the emission spectra of the protein samples in the presence of increasing concentrations of thyroxine.

In addition to fluorescence and CD, refolded species of WT- and V30M-TTR were characterized by size-exclusion chromatography (SEC) (Figure 3A,C) and thyroxine binding assays (Figure 3B,D). SEC chromatograms show the presence of one major peak (>95%), with an elution volume of approximately 22 mL and an apparent molecular mass of 55 kDa, clearly demonstrating the tetrameric nature of the refolded species of WT- and V30M-TTR. The elution volume of these refolded species is exactly the same as the one observed for native tetrameric TTR (Figure S1). Furthermore, the absence in the chromatogram of other molecular species, in any significant amount, indicates that both TTR variants refold to the tetrameric form with a very high yield. Thyroxine binding assays were performed to assess if the refolded tetramers of WT- and V30M-TTR displayed native-like binding properties. Figure 3B,D shows the isothermal binding curves of thyroxine to WT- and V30M-TTR after the refolding reaction, obtained by plotting the variation of TTR intrinsic fluorescence intensity as a function of thyroxine concentration. The fluorescence spectra of the refolded tetramers of WT- and V30M-TTR (inset graphics in Figure 3B,D) show quenching in fluorescence upon thyroxine addition. This decrease in fluorescence intensity is most likely due to the deactivation of the TTR fluorophores’ excited state by the iodine atoms of thyroxine. Several synthetic TTR ligands lacking halogen atoms do not quench the TTR fluorescence upon binding, which evidences the role of the iodine atoms in the mechanism of fluorescence quenching [34]. Because no shifts in the fluorescence emission maxima are observed, no major conformational changes are expected to occur in TTR upon thyroxine binding, which is also evident from the analysis of the crystal structures of TTR in the absence and presence of thyroxine [43].

The apparent association constants (*K_a_*) for thyroxine binding were determined by fitting Equation (1) to the experimental data. For native WT-TTR and its amyloidogenic variant V30M-TTR, the apparent *K_a_* values obtained were (1.6 ± 0.2) × 10^6^ M^−1^ and (1.5 ± 0.4) × 10^6^ M^−1^, respectively, while for the refolded tetramers, the constants were (1.5 ± 0.3) × 10^6^ M^−1^ and (1.3 ± 0.4) × 10^6^ M^−1^, respectively. The similarity between the thyroxine association constants for native and refolded TTR indicates that both WT- and V30M-TTR refold to the active tetrameric form.

### 2.4. Monomer to Tetramer Refolding Kinetics

TTR refolding from urea denaturing conditions is associated with a significant change in the tryptophan residues environment, as revealed by a large blue-shift in the emission maximum of approximately 15 nm (Figure 2A,C), from 355 nm (emission maximum characteristic of unfolded monomers) to about 340 nm (emission maximum characteristic of native tetramers). Thus, fluorescence spectra taken at intermediate urea concentrations were also collected in order to better characterize the spectroscopic nature of the protein species involved in the refolding mechanism and to better identify transition regions (as shown in Figure S2). Since tryptophan residues are located at the inter-monomer and inter-dimer interface of TTR, monitoring intrinsic tryptophan fluorescence changes seems to be an appropriate methodology to measure the folding and assembly kinetics of the native TTR tetramer. Refolding kinetics of WT- and V30M-TTR was followed by changes in intrinsic fluorescence at 380 nm upon tryptophan excitation at 290 nm.

**Figure 4:**
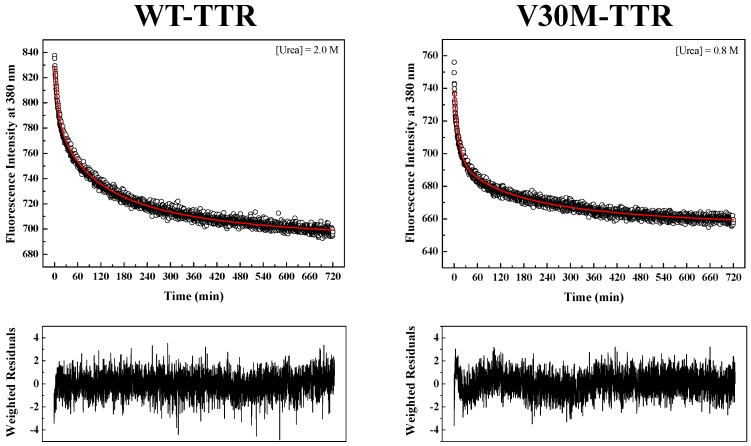
Refolding kinetics of WT-TTR and V30M-TTR monitored by intrinsic fluorescence emission. Fluorescence intensity decays (**upper panels**) for WT- and V30M-TTR monitored at different urea concentrations, pH 7.0 and 25 °C. The best fitting curves (red lines) to the experimental data points were obtained using Equations (3) to (6). Refolding assays were performed at constant protein concentrations (1.0 µM). Intrinsic fluorescence was monitored at 380 nm with an excitation wavelength of 290 nm. **Lower panels** show weighted residuals.

TTR refolding was initiated by the dilution of solutions of unfolded TTR, containing 6.0 M urea, to the desired final urea and protein concentrations. Figure 4 shows two examples of fluorescence intensity decays obtained for WT- and V30M-TTR refolding. In order to fit the data, several kinetic models were tested, among them kinetic models based on a single-step (*U* → *T*) and on two-step (*U* → *I* → *T*) refolding mechanisms. Data were initially fit to kinetic models based on a single-step mechanism (simulating first-order and second-order reactions), but the fits were of poor quality and were discarded (Figure S3). Consequently, more complex mechanisms, based on a two-step model (*U* → *I* → *T*), were attempted with more success. Good fits to the data were obtained with a two-step mechanism with two consecutive first-order reactions, but the rate constants obtained showed dependence on protein concentration (Figure S4A), indicating that higher order reactions have to be considered. Additionally, although previous fluorescence experiments have led us to propose a two-step mechanism of two consecutive bimolecular second-order steps [44], taking into account the monomeric nature of the intermediate species identified by SEC and DGGE (see below), a two-step model (*U* → *I* → *T*) involving a first-order reaction followed by a second-order reaction was tried. Fits of the experimental data were very good, with reduced weighted residuals and with no protein concentration dependencies of the rate constants (Figure S4B).

**Figure 5:**
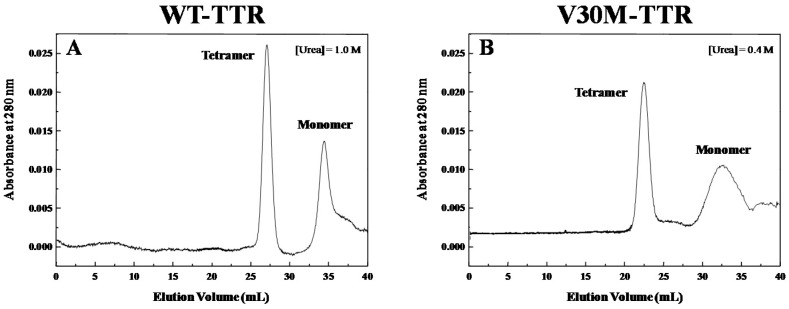
Characterization of the TTR intermediate species. Size-exclusion chromatograms of WT-TTR (**A**) and V30M-TTR (**B**) after dilution-induced protein refolding, in the presence of low concentrations of urea. SEC experiments were run at a flow rate of 0.4 mL/min, in 20 mM sodium phosphate buffer, 150 mM sodium chloride, pH 7.0, at 25 °C, in the presence of 1.0 M and of 0.4 M urea, for WT- and V30M-TTR, respectively. Prior to SEC, protein samples were submitted to 2.0 M GdmSCN for 12 h and then dialyzed against the chromatography buffer. In both chromatograms, the two main peaks can be assigned to tetramers and monomers with apparent molecular weights differing by about four-fold.

In order to investigate the nature of the intermediate species involved in the refolding process of TTR, we conducted SEC experiments, as shown in Figure 5. These experiments were run at a relatively low concentration of denaturant, favoring the occurrence and accumulation of intermediate species in solution upon partial refolding. SEC chromatograms (Figure 5) show only two main populations of protein species in solution at low denaturant concentration. Peaks at earlier elution times are attributed to the refolded forms of tetrameric WT- and V30M-TTR (apparent molecular weights of 50 to 60 kDa), whereas peaks with later elution times are consistent with apparent molecular weights of TTR monomers (11 to 16 kDa). Understandably, while the elution volume registered for tetrameric V30M-TTR remains unchanged (approximately 22 mL) and is equal to the volume observed when a sample of tetrameric WT-TTR is loaded onto the column (Figure S5), the elution volume for WT-TTR in the presence of 1.0 M urea is slightly larger (approximately 27 mL) due to the considerable increase in viscosity of the mobile phase, caused by the presence of urea in the equilibrating buffer (20 mM sodium phosphate buffer, 150 mM sodium chloride, 1.0 M urea, pH 7.0). Likewise, the elution volumes detected for the monomeric species are also slightly different in the two chromatograms, namely 32 mL for V30M-TTR (Figure 5B) and 35 mL for WT-TTR (Figure 5A). Additionally, the broader peaks observed for the monomeric species, in particular for V30M-TTR, may reflect the less compact nature of these molecular species and exchange between multiple monomeric conformations.

Thus, as suggested by the DGGE experiments (Figure 1), these results not only point towards the existence of a refolding intermediate, but also suggest that the intermediate species exhibit a monomeric nature.

**Figure 6:**
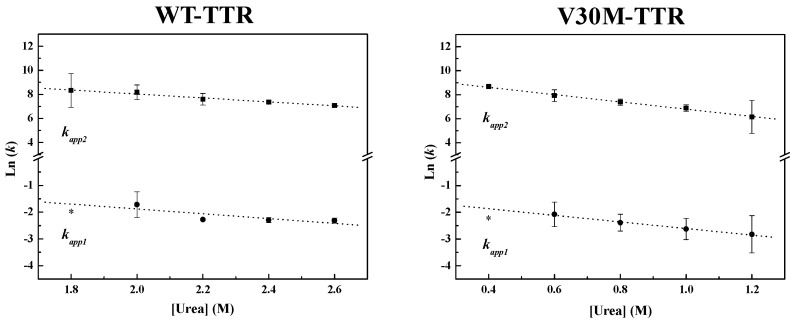
Urea dependence of the WT-TTR and V30M-TTR apparent refolding rate constants, measured by intrinsic fluorescence at 380 nm. Plots of the apparent refolding rate constants (*k_app_*) of WT- and V30M-TTR against urea concentration, obtained at 1.0 µM protein concentration, pH 7.0 and 25 °C. The plotted rate constants and standard deviation (SD) error bars are an average of at least three independent experiments. Symbols (●) and (■) represent the values of *k_app_*_1_ and *k_app_*_2_ at each urea concentration, respectively. Dashed lines are the linear least-squares fits for *k*_1_ and *k*_2_ using Equation (7). * Although values for *k_app_*_1_ at 0.4 M urea for V30M-TTR and 1.8 M urea for WT-TTR have also been determined, they were not taken into account for the extrapolation of *k*_1_, since at these concentrations of urea, the initial phase of the exponential decays was too fast to be measured accurately.

In order to obtain kinetic constants independent of protein concentration, a two-step kinetic mechanism based on a first-order reaction followed by a second-order reaction had to be postulated (Figure S4B). As shown in Figure 4, the weighted residuals of the fits, using equations representing such a mechanism, exhibit small dispersion, which indicates that the chosen kinetic model is appropriate to describe the refolding mechanism of the TTR variants under analysis. The apparent refolding rate constants for WT- and V30M-TTR, at different final urea concentrations, are shown in Table 1. The extrapolation of the experimentally-determined apparent rate constants (*k_app_*) to conditions of absence of urea, allows the direct comparison of the folding rates of WT- and V30M-TTR. Plots of the apparent rate constants *k_app_*_1_ and *k_app_*_2_ as a function of urea concentration are shown in Figure 6. Each data point is an average of at least three independent measurements, and a linear least-square fit of Equation (7) to the data yields kinetic constants in the absence of urea (Table 1). Rate constants for V30M-TTR refolding in the absence of urea are lower than those for WT-TTR: *k*_1_ is four-fold lower, and *k*_2_ is five-fold smaller. In addition, there is no experimental evidence to suggest that the TTR refolding pathway may include rate-limiting processes, such as isomerization [45], because in general, these kinetic steps present weak or even no denaturant concentration dependence.

**Figure 7:**
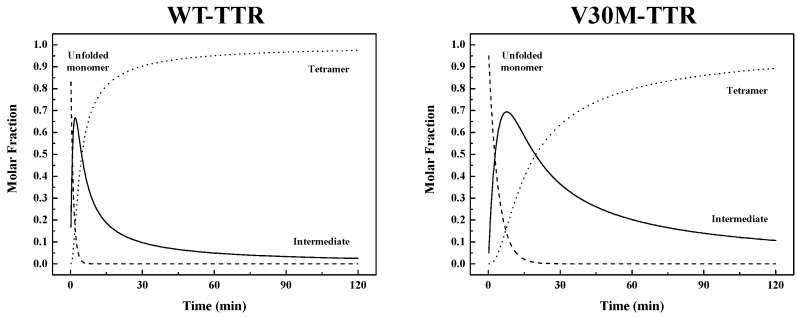
Simulation of molar fractions of TTR protein species*.* The three protein species (*U*, unfolded monomer (dashed lines); *I*, intermediate (solid lines); *T*, tetramer (dotted lines)) participate in the two-step refolding process (*U* → *I* → *T*) of TTR over time, as predicted by the rate constants (*k*_1_ and *k*_2_) obtained by extrapolation to 0.0 M urea (Equation (7); Table 1). Although the initial rate of conversion of urea-denatured TTR monomers into the corresponding intermediate is fast for both proteins, while unfolded monomers of WT-TTR are completely consumed within less than 5 min, the same process takes longer than 15 min for V30M-TTR. Additionally, whereas in the case of WT-TTR, the intermediate is the major species between 45 s and 4.6 min of reaction time, in the case of V30M-TTR, this occurs between 2.7 and 19.6 min.

Results indicate that the refolding mechanism that better fits our experimental data, as concerns WT- and V30M-TTR, involves two steps and the presence of an intermediate. The data also show that the amyloidogenic variant V30M-TTR refolds about five-times slower than WT-TTR (Table 1). Thus, the refolding process from unfolded monomers to the corresponding native homotetramer is kinetically more favorable for WT-TTR than for V30M-TTR. The rate constants emerging from the best fits were used to simulate the molar fractions of all protein species (*U*, *I* and *T*) over time, extrapolated to a 0.0 M urea concentration, for WT- and V30M-TTR (Figure 7). Analysis of Figure 7 shows that the refolding process of WT-TTR occurs within a 2 h period, whereas for the amyloidogenic variant V30M-TTR (Figure 7), the refolding process is only complete after more than 10 h. More importantly, the time the intermediate species persist in solution is significantly longer for V30M-TTR. While for WT-TTR, the intermediate appears and is reduced to less than 10% in less than 30 min, for V30M-TTR after 2 h, the intermediate is still above 10% (Figure 7).

## 3. Discussion

Most amyloid diseases are caused by the accumulation of extracellular cytotoxic protein aggregates produced by non-native protein conformations [1,2,9,10]. Therefore, issues like protein conformational stability and protein unfolding and refolding kinetics, which determine the ensemble of non-native partial unfolded conformations accessible to a protein, may be crucial for amyloidogenesis [11]. In the case of TTR, a tetrameric protein, additional factors like tetramer dissociation kinetics [30] and subunit conformational stability [29] have been shown to play a role.

Herein, we compare the refolding kinetics of the tetrameric protein transthyretin (WT-TTR) with the refolding kinetics of one of its most common amyloidogenic variants, V30M-TTR. Experiments were carried out at relatively low protein concentrations, within the observed physiological levels, which range between 3.6 μM in the plasma and 0.31 μM in the cerebral spinal fluid [24]. Moreover, we have also conducted our experiments at physiological-like ionic strengths and pH values, and we directly monitored the intrinsic fluorescence properties of the protein species in solution, i.e., protein species involved in the refolding mechanism. Since TTR has two tryptophan residues per subunit, located at the interfaces monomer-monomer and dimer-dimer, changes in intrinsic fluorescence may report not only the folding, but also the reassembly of the TTR tetramer. Thus, we have monitored protein refolding from urea-unfolded WT- and V30M-TTR, by following intrinsic fluorescence changes, in order to compare both proteins and find out how the presence of the V30M mutation influences the folding kinetics of these two TTR variants, with different amyloidogenic potentials in vivo. Denaturant gradient gel electrophoresis experiments were also performed to provide complementary information about the refolding transition from urea-induced unfolded TTR. This technique is particularly sensitive to sample heterogeneity, because molecules that differ in their electrophoretic mobilities, either in the native or unfolded states, can be easily identified. DGGE experiments showed that mutations interfere with the refolding behavior of TTR, with the reassembly of the tetramer of the amyloidogenic variant V30M-TTR observed at lower urea concentrations. Furthermore, although V30M-TTR refolds at a slower rate than WT-TTR, both TTR variants share the same refolding kinetic mechanism as demonstrated by the best fits obtained for a two-step/three-state mechanism for folding and assembly of WT- and V30M-TTR (Figure 4).

The folding mechanisms of a number of other homotetrameric proteins have already been studied by other authors, and in many cases, folding and assembly were postulated to occur via an unfolded monomer (*U*), monomeric intermediate (*I*), tetramer (*T*) pathway (*UIT* mechanism). Among these are peanut agglutinin [46], pyrrolidone carboxyl peptidase [47] and potassium channel KcsA [48], just to give some examples. Additionally, the two-step refolding mechanisms exhibited by these proteins [46,47,48] also occur through a first fast step, associated with conformational rearrangement of monomers, followed by a second slower step, associated with the final assembly of the tetrameric units.

Kelly and collaborators have previously studied the refolding kinetics of WT-TTR by following tetramer assembly indirectly, via the binding of an extrinsic fluorescent compound and using a wide range of TTR concentrations (0.72 to 36 μM in monomer) [37]. To fit the data over such a wide protein concentration range, the authors postulated a more complex and unusual folding mechanism involving a monomer (*M*), dimer (*D*), trimer (*R*), tetramer (*T*) pathway (*MDRT* mechanism). However, the authors have also suggested that at low protein concentrations, simpler mechanisms could occur [37]. Furthermore, it has been pointed out that while for the *UIT* mechanism, the tetramer is inevitably the end product, this is not necessarily true for the *MDRT* mechanism, since the protein may be trapped in intermediate oligomeric states [49]. Our kinetic data fit very well (with very low weighted residuals) the conventional *UIT* mechanism, in accordance with what has been postulated for other homotetrameric proteins [46,47,48]. Thus, the refolding mechanisms of WT- and V30M-TTR, at physiologically-relevant protein concentrations, comprise a single intermediate, most likely monomeric in nature, stable enough to accumulate under the experimental conditions tested. The formation of this intermediate correspond to an initial fast step represented by the first kinetic constant *k*_1_, while the second slower step, represented by the kinetic constant *k*_2_, corresponds to the final assembly of the homotetramer. If any additional steps exist, such as the formation of dimers or conformational rearrangements before the native state is reached, they should be either very rapid, and hence, appear coupled to the reassembly process, or spectroscopically silent and not observed by changes in protein intrinsic fluorescence, DGGE or SEC.

In sum, despite the common refolding mechanism that WT- and V30M-TTR seem to share, refolding of the WT protein is significantly faster. Extrapolation of the refolding constants to 0.0 M urea (Table 1) shows that the first step in the refolding mechanism is approximately four-times faster for WT-TTR, whereas the second step is almost five-times faster. These differences in the refolding constants are translated into differences in global refolding times with WT-TTR *t*_90%_ of 30 min, four-times faster than V30M-TTR with a *t*_90%_ of 120 min (with *t*_90%_ = time to reach a 0.9 molar fraction of tetramers). Hence, for WT-TTR, the intermediate monomer is the major species between 45 s and 4.6 min of reaction time, while in the case of V30M-TTR, this occurs between 2.7 and 19.6 min (Figure 7). On a qualitative basis, the results of urea gradient gel electrophoresis experiments also support this conclusion, since the refolding transition of V30M-TTR observed in our study appears to be slower than WT-TTR, on the time scale of the electrophoresis, given that such differences in the kinetics of transition can be inferred from the lack of continuity of the protein electrophoretic bands. In fact, several studies have shown that a sharp continuous band generated through the transition zone is typical of a rapid folding equilibrium, whereas a smeared or discontinuous band indicates a slow transition, within the time of electrophoresis [38,39,40,41].

Studies conducted with other amyloidogenic proteins have also shown that even small differences in the magnitude of the kinetic constants for protein refolding between amyloidogenic variants and their wild-type counterparts can justify different tendencies for aggregation [50,51,52]. Knowing that in most amyloidoses, amyloid deposition occurs extracellularly and might be a consequence of unfolding-misfolding events, the much slower refolding times presented by the amyloidogenic variant V30M-TTR may be of critical importance for amyloid formation in vivo. The slower rate at which tetramer assembly occurs for the amyloidogenic variant may facilitate the accumulation of monomers in the extracellular environment, which may in turn result in a preferential kinetic partition into aggregation pathways instead of the native refolding pathway [32].

## 4. Materials and Methods

### 4.1. Materials

Guanidinium thiocyanide (GdmSCN), urea, acrylamide, thyroxine (T_4_) and all other chemicals were of the highest purity commercially available and were purchased from Sigma-Aldrich Co (St. Louis, MO, USA).

### 4.2. Protein Sample Preparation

Recombinant WT- and V30M-transthyretin were produced in an *Escherichia coli* expression system [53] and purified as described previously [54]. Protein concentrations were determined spectrophotometrically at 280 nm, using an extinction coefficient of ε_280_ (1%) = 14.1 mg^−1^·mL·cm^−1^, based on a molecular mass of 55 kDa for TTR [55]. Protein samples were prepared in 20 mM sodium phosphate buffer, 150 mM sodium chloride, at pH 7.0.

### 4.3. Transthyretin Denaturation Procedure

Tetrameric TTR is highly stable and difficult to denature even at high urea concentrations [56]. Therefore, in order to avoid long exposure times (24 to 72 h) to high urea concentrations required for TTR unfolding that may lead to chemical modification of the protein [57,58], chemically-induced unfolding of TTR was accomplished in two steps. Firstly, native protein samples were incubated in 2.0 M GdmSCN for 12 h, followed by dialysis against 6.0 M urea during 10 h. Once tetramer dissociation and unfolding were achieved by the action of GdmSCN, urea was used, as a second chaotropic denaturant, in order to maintain the fully-denatured state (only with the presence of monomers in solution). Denaturant solutions were prepared in 20 mM sodium phosphate buffer, 150 mM sodium chloride, at pH 7.0. Stock solutions of GdmSCN were prepared at an approximate concentration of 5.8 M. Freshly-prepared 6.0 M urea stock solutions were employed in all experiments. The concentration of stock solutions of urea were verified by their refractive index [59], with measurements performed on an Automatic Refractometer (Rudolph Research Analytical, Flanders, NJ, USA). Once the full denaturation of the TTR tetramers into monomers was accomplished, as described above, the protein samples were refolded by dilution into 20 mM sodium phosphate buffer, 150 mM sodium chloride, pH 7.0, to the desired urea and protein concentrations, at 25 °C. The protein refolding process was followed by urea gradient gel electrophoresis and intrinsic fluorescence.

### 4.4. Refolding Experiments

#### 4.4.1. Denaturant Gradient Gel Electrophoresis

Transverse urea gradient gels were prepared as described previously, with minor modifications [38]. Urea gradient polyacrylamide gels were prepared in Tris-acetate buffer (50 mM, pH 7.5), with a horizontal linear gradient of urea concentration and a constant acrylamide concentration of 9% (acrylamide-bisacrylamide ratio 49:1). Polymerized gels were stored at 4 °C and used within 48 h. The 8.2 cm × 10.1 cm gel plates with spacers 1 mm thick were turned 90° degrees for electrophoresis. Before applying the protein samples, the gels were pre-electrophoresed for 30 min to remove any reactive compounds that could originate artefacts. Either native or previously denatured TTR samples (WT- and V30M-TTR), at approximately 1.0 μM as tetramer, were applied across the top of the gel. Electrophoresis was typically performed at a constant current of 20 mA for 4 h. Protein bands in the gel were visualized by silver staining [60]. The experiments performed by denaturant gradient gel electrophoresis (DGGE) were repeated at least for three independent times.

#### 4.4.2. Intrinsic Tryptophan Fluorescence

The refolding process, from fully-denatured monomers into native tetramers, was also monitored by intrinsic tryptophan fluorescence, since the TTR variants under study present two tryptophan residues per monomer (located at positions 41 and 79). The refolding reaction was allowed to proceed for 720 min (12 h). Protein refolding experiments were carried out at several urea concentrations. To determine the apparent refolding rate constants of WT-TTR and the amyloidogenic variant V30M-TTR, final concentrations of urea varied between 1.8 and 2.6 M (for WT-TTR) and 0.4 to 1.2 M (for V30M-TTR) and were performed using final protein concentrations raging between 0.1 μM and 1.0 μM (as tetramer). Fluorescence measurements were performed on a Varian Eclipse spectrofluorometer (Varian Ltd., Surrey, UK) equipped with a thermostated cell compartment at 25 °C, with continuous stirring. Spectra of intrinsic fluorescence of TTR were recorded in the wavelength range of 300 to 420 nm, upon excitation at 290 nm. The kinetic traces were collected at 380 nm with excitation at 290 nm, using 10- and 5-nm slit widths for emission and excitation, respectively. A 1.0 cm × 1.0 cm path length rectangular quartz cell was used for these studies. The refolding experiments were repeated at least three independent times and found to be reproducible within experimental errors.

### 4.5. Far-UV Circular Dichroism

Circular dichroism (CD) experiments were performed to monitor the structural transitions that occur during the unfolding and refolding of WT- and V30M-TTR. Far-UV CD spectra were recorded on an OLIS DSM 20 CD spectrophotometer (OLIS, Inc., Bogart, GA, USA) in the wavelength range of 190 to 260 nm and using a 0.2 mm path length cell. CD spectra of WT- and V30M-TTR were run with a step resolution of 1 nm, an integration time of 6 s and using a bandwidth of 0.6 nm. The spectra were averaged over two scans and corrected by subtraction of the corresponding buffer signal. Since solutions containing a high concentration of GdmSCN absorb too strongly in the far-UV region, the CD spectra of TTR samples incubated in this denaturant were not recorded. The absorption of urea in the far-UV also restricts the wavelength range of the CD measurements, and consequently, reliable CD spectra were only collected at wavelengths above 205 nm.

The results are expressed as the mean residue ellipticity [Θ]*_MRW_*, defined as [Θ]*_MRW_* = Θ*_obs_* (0.1*MRW*)/(*lc*), where Θ*_obs_* is the observed ellipticity in millidegrees, *MRW* is the mean residue weight, *c* is the concentration in milligrams per milliliter and *l* is the light path length in centimeters. Final spectra were smoothed using a Savitzky—Golay filter (OriginPro7 software, OriginLab Corporation, Northampton, MA, USA).

### 4.6. Size-Exclusion Chromatography

TTR samples at approximately 1.0 μM protein concentration were submitted to SEC either under physiological-like conditions (20 mM sodium phosphate buffer, 150 mM sodium chloride, pH 7.0) or under partially denaturing conditions (in the presence of 0.4 M urea for V30M-TTR and of 1.0 M urea for WT-TTR), in order to characterize the nature of the refolded species and the intermediate species, respectively.

SEC experiments were performed on an Amersham Pharmacia Biotech FPLC Superdex-75 HR column, coupled to a Pharmacia P-500 pump (Pharmacia Biotech AB, Uppsala, Sweden) and a Gilson UV detector (Gilson, Inc., Middleton, WI, USA). The column was always allowed to equilibrate with at least 5 column volumes of the chromatography buffer, prior to sample injection. Final chromatography runs were performed at a flow rate of 0.4 mL/min. Apparent molecular masses were calculated by interpolation on an elution volume versus log(molecular mass) calibration curve of four protein standards: bovine serum albumin (66.0 kDa), carbonic anhydrase (29.0 kDa), cytochrome C (12.4 kDa) and aprotinin (6.5 kDa).

### 4.7. Thyroxine Binding Assays

The native-like behavior of the refolded TTR samples was evaluated by the ability to bind the natural ligand thyroxine. Thyroxine binding assays were performed taking advantage of the quenching of the intrinsic protein fluorescence upon thyroxine binding. Stock solutions of thyroxine (T_4_) were prepared in 20 mM sodium phosphate buffer, 150 mM sodium chloride, pH 7.0. Concentrations of T_4_ solutions were determined spectrophotometrically at 325 nm, using an extinction coefficient of 6.18 × 10^3^ M^−1^·cm^−1^ [61]. Refolded protein samples were dialyzed against 20 mM sodium phosphate buffer, 150 mM sodium chloride, pH 7.0, before the binding assay.

Binding assays were performed by adding aliquots of freshly-prepared stock solutions of T_4_ to the refolded tetramer of TTR, at approximately 1.0 μM concentration. The final concentrations of T_4_ varied between 0.19 and 4.0 μM.

Fluorescence measurements were performed on a Varian Eclipse spectrofluorometer, at 25 °C, with continuous stirring. Intrinsic fluorescence of TTR was recorded in the wavelength range of 300 to 400 nm, upon excitation at 290 nm, using 5- and 10-nm slit widths for excitation and emission, respectively. A 0.5 cm × 0.5 cm path length rectangular quartz cell was used for these studies. The emission fluorescence data at 350 nm were analyzed by nonlinear least-squares fitting, using the software package OriginPro7 (OriginLab Corporation, Northampton, MA, USA). Experimental binding curves were fit using Equation (1):

(1) $$\Delta F_{i}=\Delta F_{\text{max}}\;\left(\frac{1+K_{a}{\left[L\right]}_{0}+nK_{a}{\left[C\right]}_{0}\;-\sqrt{\left(-4nK_{a}^{2}\;{\left[C\right]}_{0}\;{\left[L\right]}_{0}+{\left(-\;1-K_{a}\;{\left[L\right]}_{0}-nK_{a}{\left[C\right]}_{0}\right)}^{2}\right)}}{2nK_{a}{\left[C\right]}_{0}}\right)$$

where Δ*F_i_* is the variation of fluorescence intensity at each point of the titration, Δ*F_max_* is the maximum variation in fluorescence intensity, *n* is the number of equivalent binding sites, *K_a_* is the association constant and [*C*]_0_ and [*L*]_0_ are the total protein concentration and the total ligand concentration after each ligand addition, respectively [62,63].

### 4.8. Refolding Data Analysis

Refolding kinetics curves were constructed using the variation in intrinsic protein fluorescence emission at 380 nm. Several refolding kinetic schemes were tested for compatibility with the experimental data. Among the various models tested, the simplest best-fitting and more likely kinetic model for the refolding and assembly of the TTR tetramer was found to be a simple two-step mechanism:

(2) $$U\;\overset{k_{1}}{\to }\;I\;\overset{k_{2}}{\to }\;T$$

where *U*, *I* and *T* correspond to unfolded monomeric, intermediate and tetrameric species, respectively, and with the first step being first-order and the second step second-order. The system of differential equations associated with this mechanism is:

(3) $$\frac{d\left[U\right]}{dt}=\;-k_{1}\left[U\right]$$

(4) $$\frac{d\left[I\right]}{dt}=k_{1}\left[U\right]-k_{2}{\left[I\right]}^{2}$$

(5) $$\frac{d\left[T\right]}{dt}=k_{2}{\left[I\right]}^{2}$$

where the brackets indicate the molar concentration of the enclosed species and *t* is the time in s. Conservation of mass dictates that *C* = [*T*] + [*I*] + [*U*], where *C* is the total concentration of protein subunits and [*T*], [*I*] and [*U*] the concentration of subunits in tetrameric, the intermediate or the unfolded form, respectively. Since we are concerned with homotetramer assembly starting from a pool of unfolded monomers, the initial conditions are [*U*]*_t_*_=0_ = *C* and [*I*]*_t_*_=0_ = [*T*]*_t_*_=0_ = 0. Assuming these considerations, the differential equations were numerically integrated in order to obtain the concentrations of *U*, *I* and *T* over time, which were used in Equation (6). This equation describes the time course of fluorescence change, where *I_f_*(*t*) is the observed fluorescence intensity at time *t*, and *f*_T_, *f*_I_ and *f*_U_ are the specific fluorescence intensities of *T*, *I* and *U*, respectively:

(6) $$I_{f}\;\left(t\right)=f_{U}\left[U\right]\;+\;f_{I}\left[I\right]\;+\;f_{T}\left[T\right]$$

The apparent rate constants *k_app_*_1_ and *k_app_*_2_ were obtained by fitting Equations (3) to (6) to the data, using nonlinear least-squares analysis.

The dependence on urea concentration of the refolding apparent rate constants *k_app_*_1_ and *k_app_*_2_ was fit to Equation (7):

(7) $$\ln k_{app}=\ln k+m\left[Urea\right]$$

where *k_app_* is the apparent rate constant for refolding obtained at different urea concentrations, *k* is the refolding rate constant in the absence of denaturant and *m* is the refolding rate dependence on urea concentration.

## 5. Conclusions

In conclusion, here we show that, at physiologically-relevant protein concentrations, the refolding pathways of WT-TTR and one of the most frequent amyloidogenic variants V30M-TTR follow a common mechanism observed among other tetrameric proteins, i.e., a two-step mechanism with an intermediate species: unfolded monomer → intermediate → tetramer. We also show that the assembly of the native homotetramer is kinetically much more favorable for the WT protein than for the amyloidogenic variant, despite their high 3D structural similarity [26,27]. Since, in vivo, amyloidogenesis might be viewed as a question of protein stability and protein unfolding followed by kinetic partition between refolding and aggregation, the longer refolding times observed for V30M-TTR might increase the probability of the formation of non-native intermediate monomeric species prone to aggregation, which in turn would decrease the extent of native refolding. Thus, the different refolding times together with different protein concentrations in distinct physiological states may therefore be important factors to explain the selective formation of TTR amyloid fibrils, in different tissues by different TTR variants.

## Figures and Tables

**Table 1:** Refolding rate constants of WT-TTR and of its amyloidogenic variant V30M-TTR. Refolding assays were performed at constant protein concentrations (1.0 µM).

Dependence of the Refolding Rate Constants on Urea Concentration ^a^							Rate Constants Extrapolation to the Absence of Denaturant ^b^
Urea Concentration (M) ^c^							
WT-TTR		1.8	2.0	2.2	2.4	2.6	
	*k_app_*_1_^d^ (min^−1^)	*	(1.79 ± 0.81) × 10^−1^	(1.03 ± 0.03) × 10^−1^	(1.01 ± 0.09) × 10^−1^	(0.99 ± 0.09) × 10^−1^	(9.23 ± 3.23) × 10^−1^
	*k_app_*_2_^e^ (M^−1^·min^−1^)	(4.11 ± 3.69) × 10^3^	(3.53 ± 2.07) × 10^3^	(1.96 ± 0.68) × 10^3^	(1.57 ± 0.28) × 10^3^	(1.19 ± 0.22) × 10^3^	(8.22 ± 0.28) × 10^4^
V30M-TTR		0.4	0.6	0.8	1.0	1.2	
	*k_app_*_1_^d^ (min^−1^)	*	(1.32 ± 0.59) × 10^−1^	(0.95 ± 0.31) × 10^−1^	(0.77 ± 0.30) × 10^−1^	(0.67 ± 0.43) × 10^−1^	(2.56 ± 0.03) × 10^−1^
	*k_app_*_2_^e^ (M^−1^·min^−1^)	(5.84 ± 0.91) × 10^3^	(2.96 ± 1.42) × 10^3^	(1.64 ± 0.46) × 10^3^	(1.01 ± 0.26) × 10^3^	(0.70 ± 0.74) × 10^3^	(1.84 ± 0.01) × 10^4^

^a^ Refolding rate constants were obtained by fitting Equations (3) to (6) to the experimental data, at different urea concentrations; ^b^ refolding rate constants (*k*) of both TTR variants in the absence of denaturant were obtained by extrapolation of the refolding rates at different urea concentrations using Equation (7) (see Figure 6); ^c^ final urea concentration in the refolding mixture; ^d^ *k_app_*_1_, apparent first-order rate constant for the initial step of the two-step refolding mechanism, corresponding to the formation of a refolding intermediate; ^e^ *k_app_*_2_, apparent second-order rate constant for the slower step of the refolding mechanism, corresponding to the formation of the native TTR homotetramer; * although values for *k_app_*_1_ at 0.4 M urea for V30M-TTR and 1.8 M urea for WT-TTR have also been determined, they were not taken into account for the extrapolation of *k*_1_, since at these concentrations of urea, the initial phase of the exponential decays was too fast to be measured accurately.

## Supplementary Materials

Supplementary materials can be found at www.mdpi.com/1422-0067/17/9/1428/s1.

## Abbreviations

TTR	transthyretin
ATTR	TTR-related amyloidosis
FAP	familial amyloidotic polyneuropathy
WT	wild-type
V30M-TTR	transthyretin with Val at position 30 replaced by Met
GdmSCN	guanidinium thiocyanide
T_4_	thyroxine
DGGE	denaturant gradient gel electrophoresis
CD	circular dichroism
SEC	size-exclusion chromatography
*U*	unfolded monomer
*I*	intermediate
*T*	tetramer
