# Supplementary material for: A New Folding Kinetic Mechanism for Human Transthyretin and the Influence of the Amyloidogenic V30M Mutation
Source: Int J Mol Sci. 2016 Aug 31;17(9):1428. doi: 10.3390/ijms17091428 (PMC5037707; doi:10.3390/ijms17091428)
Supplement: Supplementary file 1 [file ijms-17-01428-s001.pdf]

Supplementary Materials: A New Folding Kinetic Mechanism for Human Transthyretin and the Influence of the Amyloidogenic V30M Mutation

Catarina S. H. Jesus, Zaida L. Almeida, Daniela C. Vaz, Tiago Q. Faria and Rui M. M. Brito

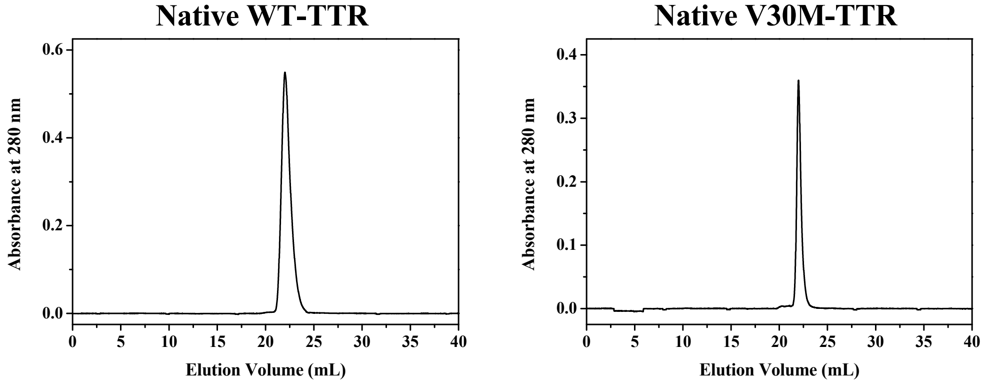

Figure S1. Characterization of the TTR native species. Size-exclusion chromatograms of WT-TTR and V30M-TTR in native conditions (20 mM sodium phosphate buffer, 150 mM sodium chloride, pH 7.0) and 25 °C. The single peak with an elution volume of approximately 22 mL and a molecular weight of nearly 55 kDa corresponds to the native tetrameric form of TTR.

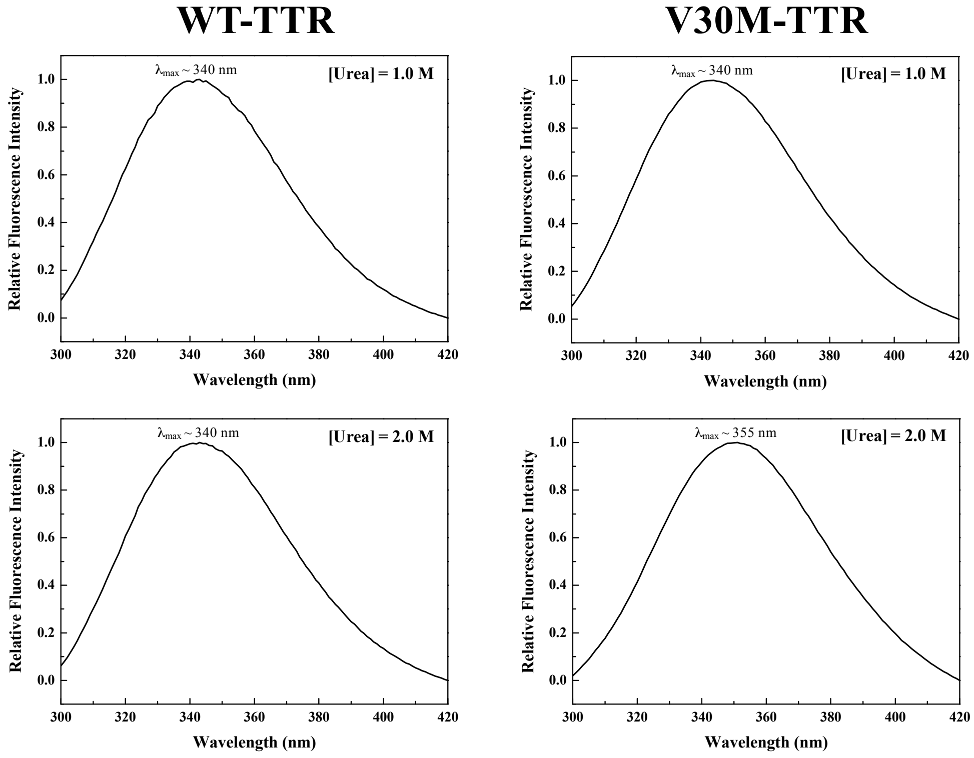

Figure S2. Fluorescence spectra of WT-TTR and V30M-TTR at intermediate urea concentrations. Fluorescence spectra of 1.0 μM WT-TTR and V30M-TTR obtained upon dialysis of fully-unfolded protein samples against 1.0 M and 2.0 M urea (in 20 mM sodium phosphate buffer, 150 mM sodium chloride, pH 7.0), evidencing differences in emission maxima (with excitation wavelength of 290 nm). While at 1.0 M urea, both WT- and V30M-TTR show emission maxima close to 340 nm (characteristic of native tetramers), which is not the case at 2.0 M urea, where only WT-TTR exhibits the emission spectrum characteristic of native tetrameric TTR.

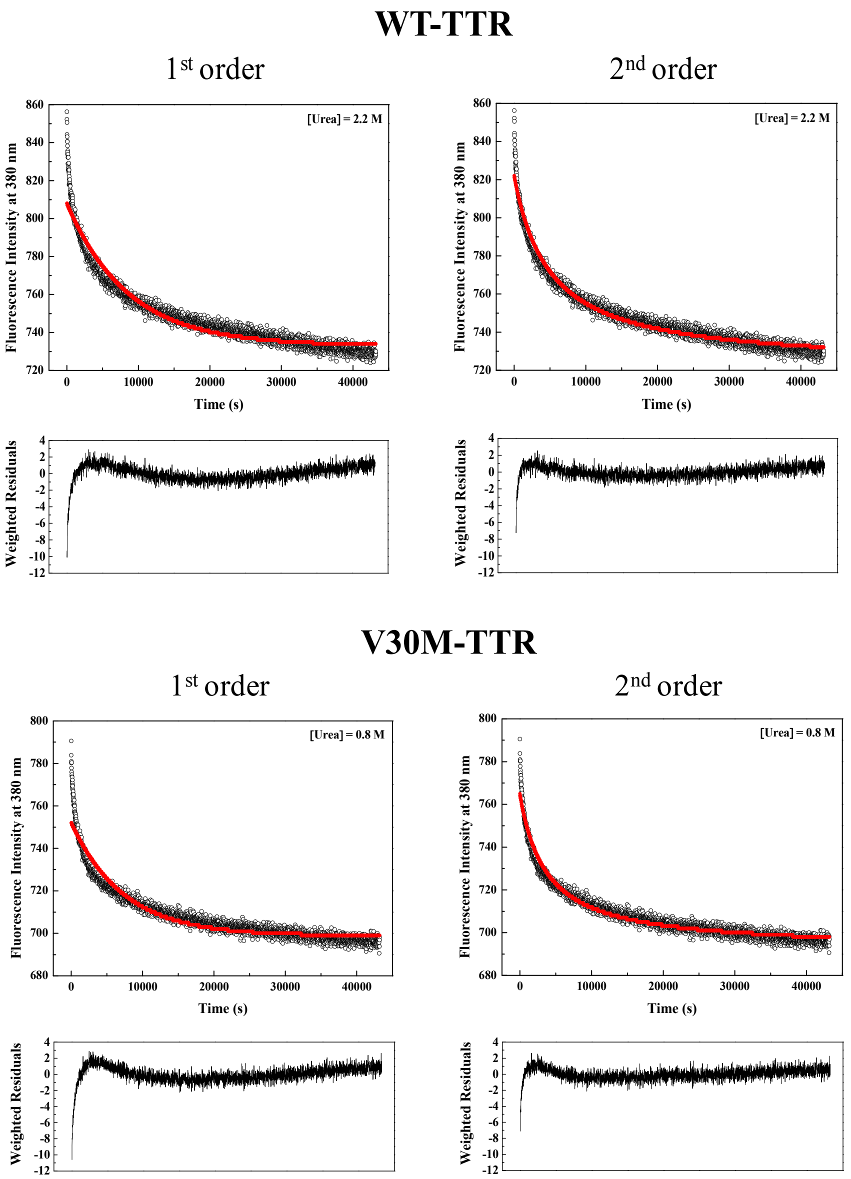

Figure S3. Fits of TTR refolding traces using a single-step model. Fluorescence intensity decays (**upper panels**) and weighted residuals (**lower panels**) obtained for WT- and V30M-TTR at different urea concentrations, pH 7.0 and 25 °C, at a 1.0 μM protein concentration. Intrinsic fluorescence was monitored at 380 nm, with an excitation wavelength of 290 nm. Data were fitted to a single-step mechanism *(U → T)*, with no intermediate species, considering first-order (**left panels**) and second-order (**right panels**) reactions. In both cases, the fits agree poorly with the experimental data, indicating that the process is likely to occur through a more complex mechanism, involving the presence of intermediate species.

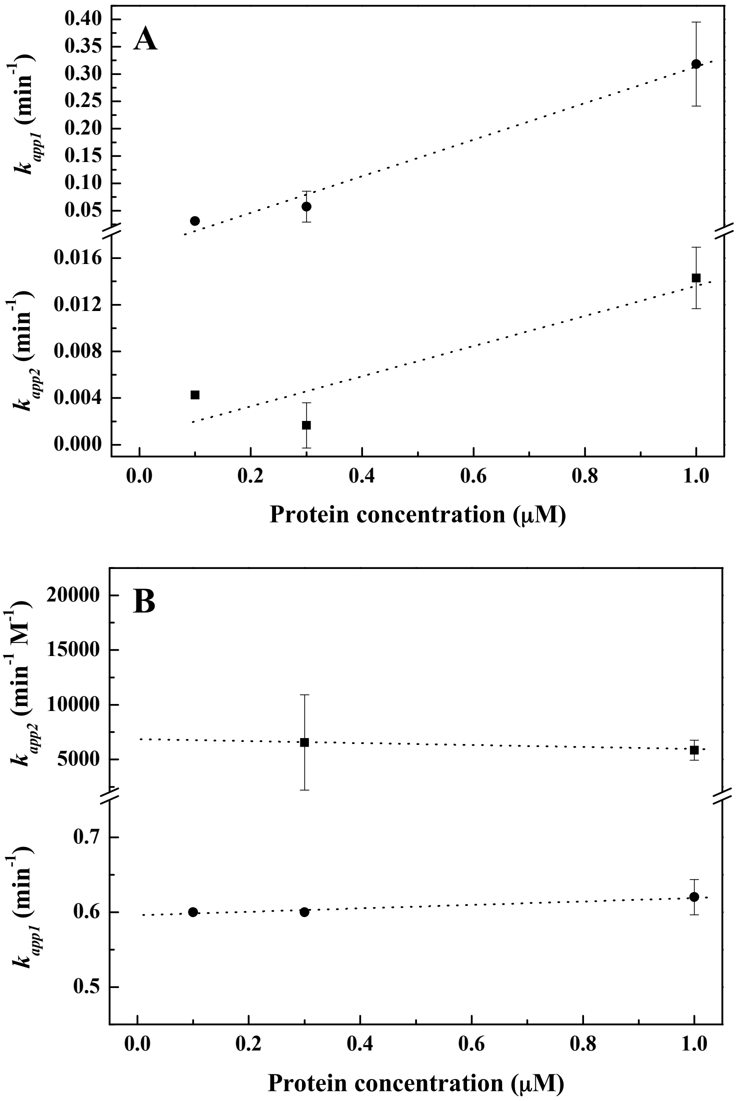

Figure S4. Dependence of the kinetic constants on TTR concentration. Apparent rate constants k_{app1} (circles) and k_{app2} (squares) at different TTR protein concentrations were obtained using a mechanism with two first-order consecutive steps (**A**) and a first-order step followed by a second-order step (**B**), as was used in the proposed mechanism in the present paper.

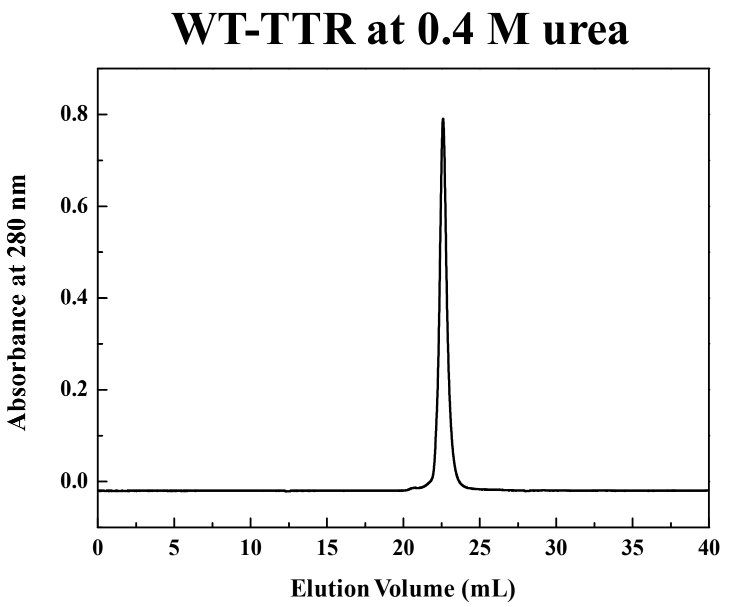

Figure S5. Size-exclusion chromatogram of WT-TTR at 0.4 M urea. Chromatogram of tetrameric WT-TTR loaded to a size-exclusion chromatography (SEC) column conveniently equilibrated with 0.4 M urea, 20 mM sodium phosphate buffer, 150 mM sodium chloride, pH 7.0, run at a flow rate of 0.4 mL/min, 25 °C. The single peak observed elutes at a volume of approximately 22 mL (corresponding to ~55 kDa, a molecular weight characteristic of native tetrameric TTR), as observed in the absence of urea.
